# Bimetallic aluminum complexes bearing novel spiro-phenanthrene-monoketone/OH derivatives: synthesis, characterization and the ring-opening polymerization of ε-caprolactone†

**DOI:** 10.1039/d1ra01288f

**Published:** 2021-04-08

**Authors:** Erlin Yue, Furong Cao, Jun Zhang, Wenjuan Zhang, Youshu Jiang, Tongling Liang, Wen-Hua Sun

**Affiliations:** Yan'an Key Laboratory of New Energy & New Functional Materials, Shaanxi Key Laboratory of Chemical Reaction Engineering, School of Chemistry and Chemical Engineering, Yan'an University Yan'an 716000 China yueerlin@yau.edu.cn; Beijing Key Laboratory of Clothing Materials R&D and Assessment, Beijing Engineering Research Center of Textile Nanofiber, School of Materials Science and Engineering, Beijing Institute of Fashion Technology Beijing 100029 China zhangwj@bift.edu.cn; Key Laboratory of Engineering Plastics and Beijing National Laboratory for Molecular Sciences, Institute of Chemistry, Chinese Academy of Sciences Beijing 100190 China whsun@iccas.ac.cn

## Abstract

A series of spiro-phenanthrene-monoketone/OH derivatives (L1–L6) were synthesized and fully characterized with ^1^H/^13^C NMR spectroscopy and elemental analyses. By treating ligands with AlMe_3_, oxygen-bridged binuclear aluminum complexes (Al1–Al6) were isolated and characterized by ^1^H/^13^C NMR spectroscopy. The molecular structures of ligands (L2, L4 and L5) and complex Al1 were determined by single crystal X-ray diffraction. In the presence of benzyl alcohol (BnOH), these aluminum complexes demonstrated high efficiency towards the ring-opening polymerization of ε-caprolactone (ε-CL), resulting in PCL in a linear manner with the BnO-end group. In addition, complexes Al1 and Al5 exhibited good catalytic activities even without BnOH. Moreover, complexes Al3 and Al6 with the bulkier substituent of ^i^Pr at the *ortho*-position of the arylamines demonstrated better catalytic activities than the analogs. Moreover, substituents on the backbone also affected catalytic behaviors.

## Abstract 2

Bimetallic aluminum complexes bearing novel spiro-phenanthrene-monoketone/OH derivatives were synthesized, and displayed good activity toward the ring-opening polymerization of ε-caprolactone.

## Graphical abstract

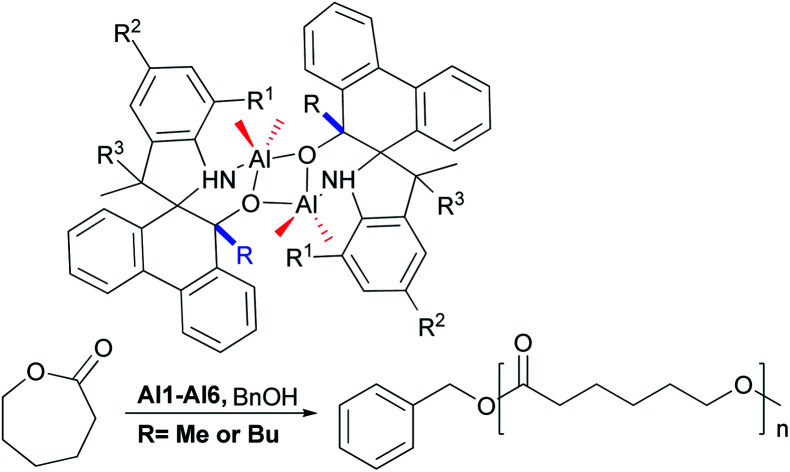

## Introduction

Biodegradable polyesters, such as polylactides (PLA) and polycaprolactone (PCL), have attracted much attention due to their biodegradability, biocompatibility and permeability, and have been extensive biomaterials in the pharmaceutical industries over the past few decades.^1^ These polyesters are commonly prepared by the ring-opening polymerization (ROP) of cyclic esters, such as ε-caprolactone (ε-CL) or lactide, and catalyzed by metal complexes as prevalent catalysts.^2^ Aluminum complex catalysts^3^ have appealed with good activities and being nicely characterized by NMR spectra in order to illustrate the coordination mechanism of the ROP of cyclic esters.^4^ Beyond ε-CL, the stereoselective ROP of *rac*-lactides was first achieved using the salen–Al complex catalyst.^5^ In general, the modifications of ligands with different substituents changed the steric and electronic environment around the metal in their aluminum complexes, thus finely tuning their activities and controllability of ROP.^6^ Attractive aluminum catalysts for ROP of cyclic esters were derived from simply N^O bidentate ligands, in which the six-membered ring N^O bidentate aluminum complexes showed less activity than their five-membered ring analogs.^7^ This was further confirmed by the DFT calculation, in which five-membered ring Al complexes reduced the steric repulsion and enhanced the activity of the ROP of ε-CL.^7^ However, there were less examples of five-membered coordinated ring Al complexes than popular six-membered ring N^O bidentate aluminum complexes.^7–9^

**Chart 1:**
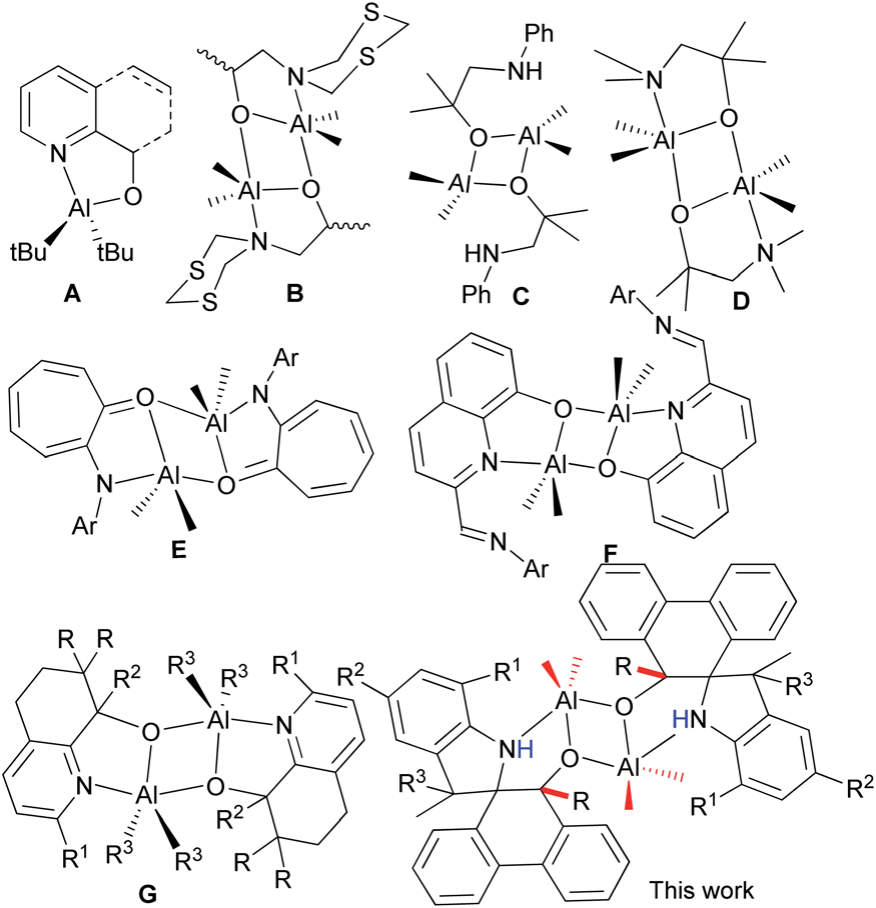
N^O bidentate aluminum complexes.

The N^O bidentate aluminum complexes (A, Chart 1) with five-membered ring coordination were verified to be in monomeric or dimeric forms that depended on the bulkiness of Al–R,^10^ but usually formed dimeric ones (B, Chart 1) derived from 2-(1,3,5-dithiazinan-5-yl)ethanols,^11^ as well as other alcohols (C and D, Chart 1).^12^ Notably, complex D could catalyze the cycloaddition of CO_2_ with epoxies.^12*b*^ The anilinotropone-based dimeric aluminum complexes (E) demonstrated high activities in the ring-opening polymerization (ROP) of *rac*-lactides with the assistance of BnOH.^13^ Subsequently, dimeric aluminum complexes (F) initiated the ROP of ε-CL^14^ with good controllability. Meanwhile, complexes G exhibited good activity for the ROP of cyclic esters, and reflected the polymerization mechanism through NMR measurements.^15^

Phenanthrene-based ligands were widely used to coordinate with transition metals,^16^ and their metal complexes exhibited good activities for isoprene polymerization,^17^ as well as coupling reactions.^18^ However, there were a few examples with main group complexes, but with 9,10-diamido-phenanthrene only.^19^ Phenanthrenequinone derivatives react extensively with trimethylaluminum, forming novel dimeric aluminum complexes bearing spiro-phenanthrene derivatives. Interestingly, these complexes show high activities towards the ring-opening polymerization of ε-caprolactone. Herein, the synthesis and characterization of the title complexes are reported, along with their catalytic performances towards the ROP of ε-CL.

## Results and discussion

### Synthesis and characterization of the ligands (L1–L6) and aluminum complexes (Al1–Al6)

**Scheme 1:**
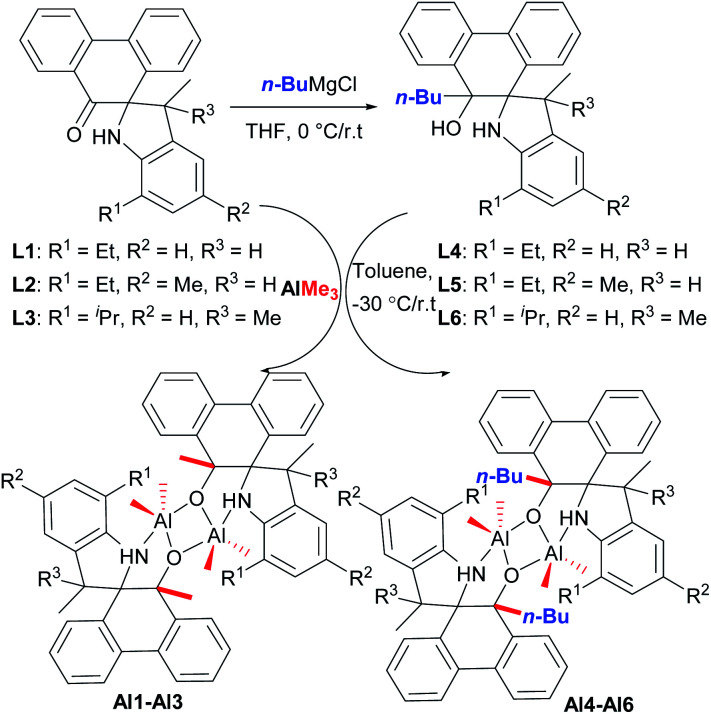
Synthesis of the spiro-phenanthrene-mono–OH derivatives L4–L6 and aluminum complexes Al1–Al6.

**Fig. 1:**
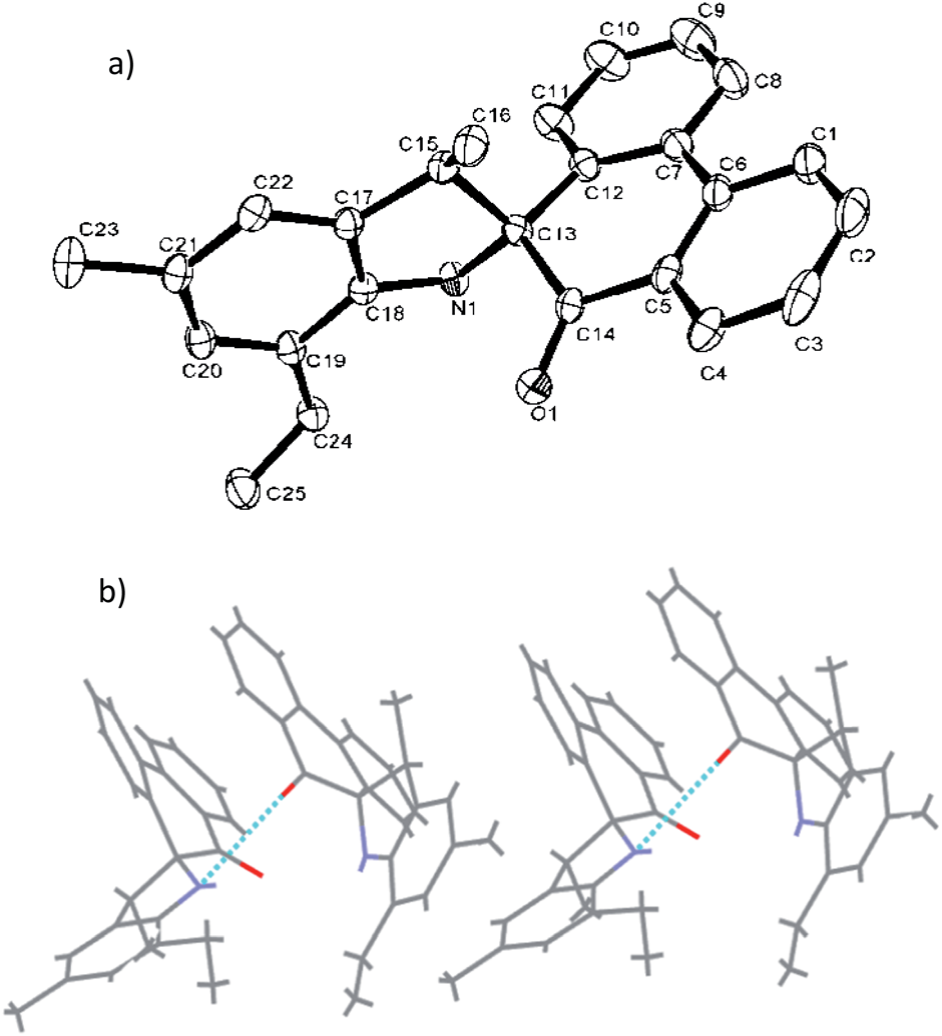
(a) ORTEP drawing of L2. Thermal ellipsoids are shown at the 50% probability level. Hydrogen atoms have been omitted for clarity. (b) Hydrogen bonding motifs in L2.

**Fig. 2:**
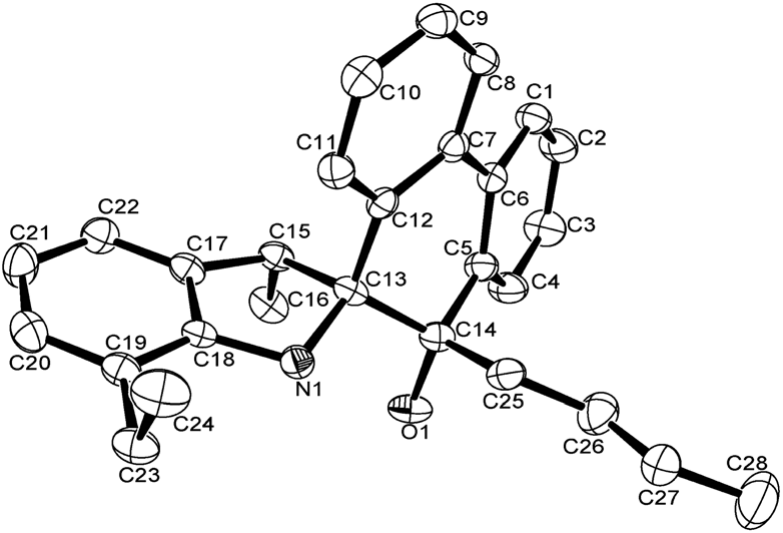
ORTEP drawing of L4. Thermal ellipsoids are shown at the 50% probability level. Hydrogen atoms have been omitted for clarity.

**Fig. 3:**
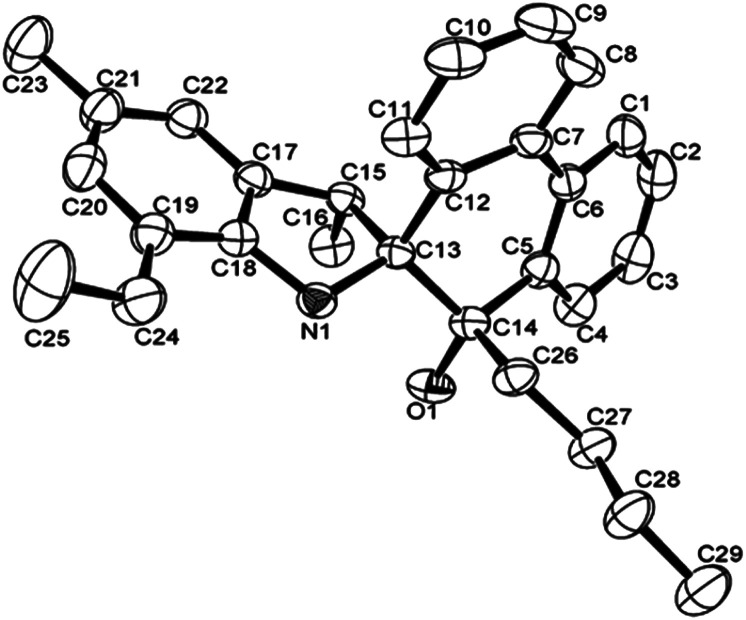
ORTEP drawing of L5. Thermal ellipsoids are shown at the 50% probability level. Hydrogen atoms have been omitted for clarity.

**Fig. 4:**
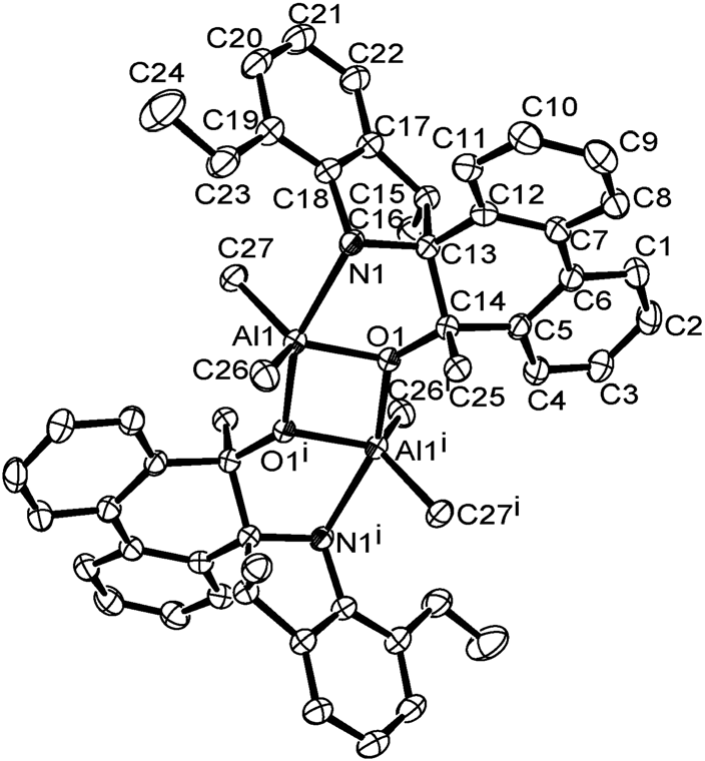
ORTEP drawing of Al1. Thermal ellipsoids are shown at the 50% probability level. Hydrogen atoms and free solvent molecules have been omitted for clarity.

According to our previous work,^18^ refluxing the mixture of 9,10-phenanthrenequinone with various substituted anilines in the presence of *p*-toluenesulfonic acid (*p*-TsOH) in toluene afforded a series of novel spiro-phenanthrene-monoketone derivatives (L1–L3). Furthermore, the reduction reaction of L1–L3 with *n*-BuMgCl gave the corresponding spiro-phenanthrene-mono–OH compounds (L4–L6) (Scheme 1). Treatment of ligands L1–L6 with one equivalent of AlMe_3_ in toluene at −30 °C gave the dialkylaluminum complexes (Al1–Al6), respectively, in acceptable yields (Scheme 1). For the preparation of Al1–Al3, one methyl group from AlMe_3_ was added to the carbonyl (C=O). For the preparation of Al1–Al6, the elimination of CH_4_ from the reaction between Al–Me and –OH occurred. All of these organic compounds and aluminum complexes were characterized by ^1^H/^13^C NMR spectroscopy and elementary analysis. Comparing the ^1^H/^13^C NMR spectra of the ligands, the disappearance of the –C=O resonance of L1–L3 and the –OH resonance of the L4–L6 in those of the corresponding aluminum complexes indicated the formation of the aluminum complexes. It is noteworthy that the resonance of –NH for all aluminum complexes Al1–Al6 still remained, being similar to the reports of the –NH presence in aluminum complexes.^8,12*a*^ Moreover, the crystal structures of the organic compounds L2, L4 and L5 and aluminum complex Al1 were determined by single crystal X-ray diffraction (Fig. 1–4).

Suitable crystals of the organic compounds L2, L4 and L5 were readily achieved from their respective heptane solution, and were determined by means of single crystal X-ray diffraction (see Fig. 1–3). Selected bond lengths and bond angles are given in Table 1.

**Table 1:** Selected bond lengths (Å) and angles (°) for L2, L4 and L5

	L2	L4	L5
**Bond lengths (Å)**			
N1–C13	1.467 (4)	1.487 (4)	1.4815 (16)
N1–C18	1.395 (4)	1.416 (4)	1.4221 (18)
O1–C14	1.218 (4)	1.427 (4)	1.4228 (16)
C13–C12	1.515 (5)	1.536 (4)	1.5356 (18)
C13–C14	1.536 (4)	1.557 (4)	1.5513 (19)
C13–C15	1.591 (5)	1.583 (4)	1.5818 (18)
C15–C16	1.525 (5)	1.537 (4)	1.5304 (19)
C15–C17	1.522 (5)	1.512 (4)	1.506 (2)
C17–C18	1.391 (5)	1.392 (4)	1.3917 (19)

**Bond angles (°)**			
C13–N1–C18	108.9 (3)	107.7 (2)	105.86 (10)
N1–C13–C12	112.8 (3)	112.7 (2)	112.06 (11)
N1–C13–C14	109.7 (3)	109.7 (2)	110.92 (10)
N1–C13–C15	102.8 (3)	102.5 (2)	102.75 (10)
N1–C18–C17	111.1 (3)	110.3 (3)	110.38 (12)
O1–C14–C5	122.4 (3)	108.0 (2)	107.02 (11)
O1–C14–C13	120.3 (3)	110.3 (3)	111.32 (11)

Fig. 1 shows the structure of L2, in which the five-membered rings are each constructed by C13, C15, C17–C18 and N1, and the O1–C14 bond length of L2 is 1.218 (4) Å, indicating a typical double-bond character of C=O. The O atom deviated from the plane of spiro-phenanthrene (C6, C2, C4, C10, C11, C8) with a distance of 0.505 Å. The plane of the aryl ring (C7, C21, C19) is almost perpendicular to the spiro-phenanthrene plane with a dihedral angle of 78.14°. From the packing model, there was hydrogen bonding of N–H⋯O between two adjacent molecules. In addition, the plane of spiro-phenanthrene is almost parallel to another phenanthrene from adjacent molecules with a small dihedral angle of 8.07°.

The structures of L4 and L5 are shown in Fig. 2 and 3 and they are similar to L2. Both possessed the five-membered rings that were constructed by C13, C15, C17–C18 and N1. However, the bond lengths of O1–C14 in L4 (1.427 (4) Å) and L5 (1.4228 (16) Å) are much longer than that of L2 (1.218 (4) Å), indicating the typical single bond characters. In addition, the bond length of N–C13, N–C18 in L4 (1.487 (4), 1.416 (4) Å), L5 (1.4815 (16), 1.4221 (18) Å) are much longer than that in L2 (1.467 (4), 1.395 (4) Å), indicating the influence of the alkyl group on C14. Due to the more distorted six-membered ring, the dihedral angle between the two phenyl rings of phenanthrene is about 19.7°, but these two phenyl rings are almost particular to the aryl ring with a dihedral angle of 76.69 and 84.1°, respectively. There were also intermolecular hydrogen bonds in the ligands L4 and L5, respectively, which came from O1–H1⋯N1. The motifs of hydrogen bonding are illustrated in Fig. S1 and S2.†

A single crystal of complex Al1 suitable for the X-ray diffraction analysis was obtained from its toluene solution. The molecular structure of complex Al1 is shown in Fig. 4, and the selected bond lengths and bond angles are collected in Table 2.

**Table 2:** Selected bond lengths (Å) and angles (°) for Al1

Bond lengths (Å)		Bond angles (°)	
Al1–O1	1.8669 (15)	O1–Al1–O1^i^	79.51 (5)
Al1–O1^i^	1.9881 (13)	O1–Al1–N1	79.59 (5)
Al1–N1	2.3291 (15)	O1^i^–Al1–N1	159.10 (5)
Al1–C26	1.9960 (18)	O1–Al1–C26	115.68 (7)
Al1–C27	1.9813 (17)	O1–Al1–C27	122.84 (7)
O1–Al1^i^	1.9882 (13)	O1^i^–Al1–C26	100.96 (6)
O1–C14	1.4504 (18)	O1^i^–Al1–C27	100.87 (7)
N1–C13	1.490 (2)	C26–Al1–C27	120.09 (8)
N1–C18	1.4388 (19)	N1–Al1–C26	88.41 (6)
Al1–Al1^i^	2.9647 (15)	N1–Al1–C27	90.18 (7)

Fig. 4 shows the complex Al1 as a dimeric form, which is symmetrically penta-coordinated by two bridged oxygen atoms (O1 and O1^i^), one nitrogen atom (N1) from the spiro-phenanthrene-monoketone ligand and two coordinating methyl anions from the trimethylaluminum. Each aluminum possessed a distorted trigonal bipyramidal geometry. Another methyl anion from trimethylaluminum was added to the carbonyl of the spiro-phenanthrene-monoketone ligand. Complex Al1 possessed a *C*_2_ symmetric axis through the centroid of the Al1–O1–Al^i^–O1^i^ plane. However, there is a slight asymmetry in the Al_2_O_2_ four-membered ring system. The bond lengths of the Al1–O1 [1.8669 (15) Å] and Al1–N1 [2.3291 (15) Å] in Al1 are shorter than the corresponding ones in the dimethylaluminum 2-chloro-6,6-dimethylcyclopentyl-pyridin-7-oxylates [1.8735 (14) Å, 2.3500 (17) Å].^15*c*^ The quadrilateral center comprises Al1, O1, Al1^i^ and O1^i^, but there is no direct bonding between the two aluminum atoms with an intramolecular distance of 2.9647 (15) Å. The dihedral angle between the two phenyl rings of spiro-phenanthrene is about 27.29°, much larger than that in L2, indicating the greater distortion of the six-membered ring after coordination with Al. In addition, although N–H remained in the Al1 complex, there was no hydrogen bonding observed in Al1 due to the bulky environment around the N atom.

### Ring-opening polymerization of ε-CL catalyzed by Al complexes Al1–Al6

First, complex Al1 was evaluated for the ring opening polymerization of *rac*-LA under various conditions, but there was no polymer product. In contrast, the ROP of ε-CL proceeded smoothly. Therefore, the ring opening polymerization of ε-CL was investigated in detail, and the polymerization results are collected in Table 3. The complex Al1 was employed to study the effect of temperature, molar ratio, and reaction time on the ROP of ε-CL. The results showed that there was no polymer observed at room temperature or 30 °C when the ROP of ε-CL was conducted at a molar ratio [CL] : [Al] : [BnOH] of [250 : 1 : 1]. When increasing the temperature from 60 °C to 90 °C (Table 3, runs 1–3), the polymer yield with 30 minutes gradually increased from 3% to 45%. Further increasing the temperature to 100 °C led to a significant increase of the polymer yield (up to 93%) (Table 3, run 4). This temperature effect on the polymerization agreed well with our previous results, in which the mononuclear species is proposed as the real active species for the ring opening polymerization of ε-CL and the ratio of the mononuclear species increased at higher temperature (verified by ^27^Al NMR).^15*b*^ However, a higher temperature of 110 °C resulted in a quick decrease of yield (64%, run 5, Table 3). The higher monomer ratio led to a slight decrease of the polymer yield and increase of the molecular weight. Prolonging the reaction time from 10 minutes to 60 minutes led to the gradual increase of yields from 89% to almost 100%. At the same time, the molecule weights are quite similar (4.28–4.86 × 10^4^ g mol^−1^), indicating the minimal effect of the reaction time. Without BnOH, Al1 still exhibited good efficiency for ROP of ε-CL with 87% polymer yield, but producing the PCL with high molecular weight (8.95 × 10^4^ g mol^−1^). When two equivalents of BnOH were used, the polymer yield was kept at 85%. However, the molecular weight sharply decreased from 8.95 to 2.55 × 10^4^ g mol^−1^, indicating the significant chain transfer of BnOH (runs 4, 11 and 12, Table 3).

**Table 3:** The ROP of ε-CL catalyzed by complexes Al1–Al6.^a^

Run	Cat.	CL : Al : BnOH	*T*/°C	*t*/min	Isolated yields^b^ (%)	*M*_n_ cc (×10^4^)	*M*_ncald_ (×10^4^)^d^	PDI^cc^
1	Al1	250 : 1 : 1	60	30	3	1.10	0.10	1.32
2	Al1	250 : 1 : 1	80	30	26	2.05	0.75	1.44
3	Al1	250 : 1 : 1	90	30	45	1.86	1.29	1.67
4	Al1	250 : 1 : 1	100	30	93	4.37	2.66	1.41
5	Al1	250 : 1 : 1	110	30	64	3.27	1.83	1.33
6	Al1	400 : 1 : 1	100	30	85	5.48	3.89	1.39
7	Al1	250 : 1 : 1	100	10	89	4.71	2.55	1.34
8	Al1	250 : 1 : 1	100	20	91	4.28	2.60	1.51
9	Al1	250 : 1 : 1	100	40	96	4.54	2.75	1.38
10	Al1	250 : 1 : 1	100	60	>99	4.86	2.86	1.36
11	Al1	250 : 1 : 0	100	30	87	8.95	—	1.89
12	Al1	250 : 1 : 2	100	30	85	2.55	1.22	1.19
13	Al5	250 : 1 : 0	100	30	81	12.45	—	1.87
14	Al5	250 : 1 : 1	100	30	83	2.82	2.38	1.47
15	Al5	250 : 1 : 2	100	30	71	2.70	1.02	1.24
16	Al5	250 : 1 : 1	80	30	36	2.92	1.04	1.39
17	Al5	250 : 1 : 1	90	30	67	4.05	1.92	1.31
18	Al5	250 : 1 : 1	110	30	48	2.15	1.38	1.86
19	Al5	250 : 1 : 1	100	40	87	1.99	2.49	1.70
20	Al5	250 : 1 : 1	100	60	95	2.16	2.72	2.04
21	Al5	400 : 1 : 1	100	30	31	1.56	1.42	1.96
22	Al5	500 : 1 : 1	100	30	0	—	—	—
23	Al2	250 : 1 : 1	100	30	77	3.34	2.21	1.31
24	Al3	250 : 1 : 1	100	30	96	4.42	2.75	1.34
25	Al4	250 : 1 : 1	100	30	85	4.27	2.43	1.31
26	Al6	250 : 1 : 1	100	30	86	1.91	2.46	1.70

a Conditions: 20 μmol Al; 1.0 M ε-CL toluene solution.

b Isolated yield: weight of the polymer obtained/weight of monomer used.

c GPC data in THF *vs.* polystyrene standards, using a correcting factor of 0.56.^20^

d *M*_n_ (calcd) = (monomer/initiator) × (conversion) × 114 + 108 (*M*_w_ of BnOH).

Considering the potential role of the long chain of the butyl group on improving the polymerization activity or molecular weight of PCL, Al5 was also investigated for ROP under the same condition. The results showed that without BnOH, the molecular weight of the polymer by Al5 was much higher than that by Al1 (run 13 *vs.* 11, Table 3), up to 12.4 × 10^4^ g mol^−1^. When increasing the BnOH amount, the polymer yield did not change a lot. However, the molecular weight of PCL sharply decreased (runs 13–15, Table 3), also indicating the chain transfer of BnOH. In the presence of BnOH, the polymerizations were conducted at various temperatures and the highest yield was achieved at 100 °C. In prolonging the reaction time from 30 min to 60 minutes, the polymer yield gradually increased from 83% to 95%. However, upon increasing the monomer molar ratio from 250 to 400 and 500, the polymer yield dramatically decreased from 83% to 31% and 0% (runs 20–22, Table 3). It is probable that the deactivation of the complexes by impurities in the monomer account for this.

At 100 °C and with the molar ratio of [CL] : [Al] as 250 within 30 minutes, all other aluminum complexes were investigated for ROP of ε-CL. The results showed that the polymer yields by Al1–Al6 bearing the *n*-Bu group are generally lower than those by Al1–Al3 having the methyl group, which was demonstrated by the yield order: Al4 < Al1, Al5 < Al2, Al6 < Al3, indicating a steric effect of the bulkier substituent around the Al center. In addition, the R^2^ group has a small effect on the polymer yield, which was demonstrated by the same order of polymer yield: Al3 (R^2^ = H) > Al1 (R^2^ = H) > Al2 (R^2^ = Me); Al6 (R^2^ = H) > Al4 (R^2^ = H) > Al5 (R^2^ = Me). The reason is probably due to the stronger electron donor ability of methyl group.

**Fig. 5:**
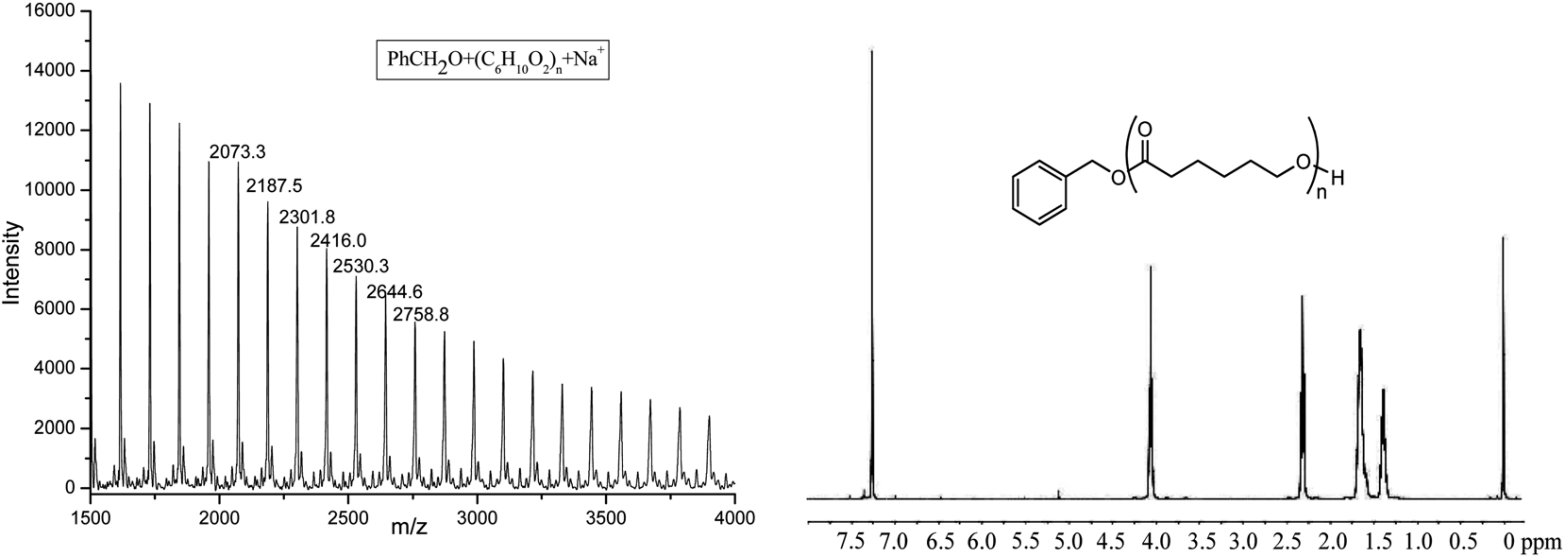
The MALDI-TOF spectrum and ^1^H NMR of PCL obtained by Al1 + BnOH (run 4, Table 3).

The obtained PCL was characterized by ^1^H NMR and MALDI-TOF spectra (shown in Fig. 5). ^1^H NMR clearly showed the signal of PhCH_2_O (7.35, 5.11 ppm), indicating the active species of Al–OCH_2_Ph in the polymerization process. The MALDI-TOF spectrum of the polymer also showed that there was one major family in the product (Fig. 5), in which the interval 114.2 Da was observed between the adjacent peaks (one repeat unit of polymer), and it can be ascribed to PhCH_2_O + 114.1 × *n* (number of repeat units) + Na^+^, a typical character of the linear structure, which agrees well with its ^1^H NMR spectrum.

## Conclusion

The oxygen-bridged binuclear aluminum complexes (Al1–Al6) bearing spiro-phenanthrene-monoketone/OH derivatives (L1–L6) were prepared and fully characterized by ^1^H/^13^C NMR spectroscopy, elemental analyses and single crystal X-ray diffraction. The crystal structure of Al1 indicated an oxygen-bridged dimeric form in the solid state. All aluminum complexes demonstrated moderate to high efficiency for the ROP of ε-caprolactone (ε-CL) in the presence of benzyl alcohol (BnOH), producing the linear PCL capped with the BnO. The complexes Al3 and Al6 containing bulkier ^*i*^Pr substitutes at the *ortho*-position of arylamine can enhance the catalytic activities for the ROP of ε-CL. More importantly, the complexes Al1 and Al5 also exhibited good catalytic activities in the absence of BnOH.

## Experimental section

### General consideration

All manipulations involving air- and moisture-sensitive compounds were performed using standard Schlenk techniques under a nitrogen atmosphere. Toluene was refluxed over sodium and distilled under nitrogen prior to use. Other reagents were purchased from Aldrich, Acros, or local suppliers. NMR spectra were recorded on a Bruker DMX 400 MHz instrument at ambient temperature using TMS as an internal standard; *δ* values were given in ppm and *J* values in Hz. ^1^H NMR spectra are referenced using the residual solvent peak at *δ* 7.26 ppm for CDCl_3_ and *δ* 7.16 ppm for C_6_D_6_. ^13^C NMR spectra are referenced using the residual solvent peak at 77.16 ppm for CDCl_3_ and *δ* 128.06 ppm for C_6_D_6_. Elemental analysis was carried out using a Flash EA 1112 micro-analyzer. Molecular weights and the molecular weight distribution (MWD) of polyethylene were determined by PL-GPC220 at room temperature, with tetrahydrofuran (THF) as the solvent. The compounds spiro-[7-ethyl-3-methylindoline-2,10-phenanthren-9-one] (L1)^18^ and spiro-[7-isopropyl-3,3-dimethylindoline-2,10-phenanthren-9-one] (L3)^16*c*^ were prepared according to the reported literature.

### Synthesis of phenanthrene-based ligands

#### Spiro-[5-methyl-7-ethyl-3-methylindoline-2,10-phenanthren-9-one] (L2)

A 100 mL toluene solution of 9,10-phenanthrenequinone (2.00 g, 9.61 mmol), 2,6-diethyl-4-methylphenylamine (3.14 g, 19.2 mmol), and a catalytic amount of *p*-toluenesulfonic acid (0.365 g, 1.92 mmol) was refluxed for 8 h. The solvent was evaporated under reduced pressure, and the mixture was then purified by silica gel column chromatography with petroleum ether/ethyl acetate (v/v = 100 : 1) as the eluent. It was then recrystallized in heptane to afford the product as a yellow powder (0.966 g) in 28% yield. ^1^H NMR (400 MHz, CDCl_3_, 25 °C): *δ* 7.97 (d, *J* = 8.0 Hz, 1H), 7.91–7.87 (m, 2H), 7.77–7.75 (m, 1H), 7.70–7.66 (m, 1H), 7.43–7.29 (m, 3H), 6.85 (s, 1H), 6.57 (s, 1H), 4.59 (s, 1H), 3.45–3.39 (m, 1H), 2.75–2.65 (m, 2H), 2.25 (s, 3H), 1.37 (d, *J* = 7.2 Hz, 3H), 0.97 (d, *J* = 7.2 Hz, 3H). ^13^C NMR (100 MHz, CDCl_3_, 25 °C): *δ* 201.5, 146.2, 143.1, 137.6, 134.7, 131.4, 131.3, 130.0, 129.9, 129.5, 128.6, 128.1, 128.0, 127.2, 127.0, 126.5, 124.2, 123.1, 121.8, 77.6, 54.7, 24.5, 21.1, 17.8, 13.8. Anal. calcd for C_25_H_23_NO: C, 84.95; H, 6.56; N, 3.96. Found: C, 84.67; H, 6.36; N, 4.01.

#### Spiro-[7-ethyl-3-methylindoline-2,10-phenanthren-9-*n*-butyl-9-hydroxyl] (L4)

Under a nitrogen atmosphere, the ligand L1 (1.00 g, 2.95 mmol) was dissolved in 30 mL THF, and then added to a solution of *n*-BuMgCl (4.4 mL, 1.0 M) in an ice bath. The mixture was stirred overnight, and then quenched by saturated ammonium chloride with stirring and extracted with CH_2_Cl_2_ (20 mL × 3). The combined organic layers were dried over anhydrous MgSO_4_, concentrated under reduced pressure, recrystallized in heptane and dried in vacuum to obtain a white powder (0.973 g) in 83% yield. ^1^H NMR (400 MHz, CDCl_3_, 25 °C): *δ* 7.74–7.67 (m, 3H), 7.41–7.27 (m, 3H), 7.13 (t, *J* = 7.2 Hz, 1H), 7.06 (d, *J* = 7.6 Hz, 1H), 6.99 (d, *J* = 6.8 Hz, 1H), 6.76–6.70 (m, 2H), 4.14 (s, 1H), 3.19–3.14 (m, 1H), 2.75–2.70 (m, 1H), 2.65 (s, 1H), 1.47–1.39 (m, 5H), 1.12 (d, *J* = 7.2 Hz, 3H), 1.07–0.95 (m, 1H), 0.79–0.71 (m, 4H). ^13^C NMR (100 MHz, CDCl_3_, 25 °C): *δ* 146.7, 144.1, 140.0, 133.5, 133.2, 128.1, 127.7, 127.6, 127.5, 126.6, 126.3, 124.7, 124.4, 123.7, 122.8, 121.9, 120.2, 77.4, 74.5, 50.4, 37.9, 25.8, 24.4, 23.1, 20.9, 14.3, 13.5. Anal. calcd for C_28_H_31_NO: C, 84.59; H, 7.86; N, 3.52. Found: C, 84.32; H, 7.92; N, 3.81.

#### Spiro-[5-methyl-7-ethyl-3-methylindoline-2,10-phenanthren-9-*n*-butyl-9-hydroxyl] (L5)

In a manner similar to that described for L4, except for using L2 instead of L1, L5 was prepared as a white powder (0.515 g) in 44% yield. ^1^H NMR (400 MHz, CDCl_3_, 25 °C): *δ* 7.73–7.67 (m, 3H), 7.40–7.28 (m, 3H), 7.12 (t, *J* = 7.6 Hz, 1H), 7.03 (d, *J* = 7.6 Hz, 1H), 6.80 (s, 1H), 6.52 (s, 1H), 4.03 (s, 1H), 3.13–3.08 (m, 1H), 2.72–2.66 (m, 3H), 2.21 (s, 3H), 1.45–1.37 (m, 6H), 1.12–1.10 (m, 4H), 0.74–0.70 (m, 4H). ^13^C NMR (100 MHz, CDCl_3_, 25 °C): *δ* 144.4, 144.3, 140.1, 133.8, 133.2, 132.1, 129.8, 128.1, 127.7, 127.6, 127.5, 127.3, 126.3, 124.7, 124.4, 123.7, 122.7, 122.6, 77.4, 74.5, 50.4, 38.0, 25.8, 24.4, 23.1, 21.2, 21.1, 14.3, 13.6. Anal. calcd for C_29_H_33_NO: C, 84.63; H, 8.08; N, 3.40. Found: C, 84.25; H, 8.21; N, 3.58.

#### Spiro-[7-isopropyl-3,3-dimethylindoline-2,10-phenanthren-9-*n*-butyl-9-hydroxyl] (L6)

In a manner similar to that described for L4, and using L3 instead of L1, L6 was prepared as a white powder (0.592 g) in 69% yield. ^1^H NMR (400 MHz, CDCl_3_, 25 °C): *δ* 7.68 (t, *J* = 7.6 Hz, 2H), 7.62–7.59 (m, 1H), 7.41–7.30 (m, 4H), 7.22–7.18 (m, 1H), 7.02 (d, *J* = 7.2 Hz, 1H), 6.75 (d, *J* = 7.2 Hz, 1H), 6.69 (d, *J* = 6.8 Hz, 1H), 4.23 (s, 1H), 3.07–3.00 (m, 1H), 2.27 (s, 3H), 1.58–1.54 (m, 2H), 1.42–1.38 (m, 6H), 1.13 (s, 3H), 1.10–0.92 (m, 2H), 0.71 (t, *J* = 7.2 Hz, 3H), 0.61 (s, 3H). ^13^C NMR (100 MHz, CDCl_3_, 25 °C): *δ* 145.0, 140.4, 140.0, 138.4, 134.6, 133.0, 128.1, 127.9, 127.8, 127.7, 127.5, 126.3, 125.6, 124.0, 123.9, 123.3, 119.7, 119.5, 79.5, 78.2, 50.2, 38.1, 29.7, 29.3, 27.8, 25.9, 23.1, 22.4, 14.2. Anal. calcd for C_30_H_35_NO: C, 84.66; H, 8.29; N, 3.29. Found: C, 84.45; H, 8.31; N, 3.42.

### Synthesis of aluminum complexes Al1–Al6

#### Synthesis of Al1

Into a stirred solution of compound L1 (0.34 g, 1.0 mmol) in toluene (10 mL), 1.0 mL AlMe_3_ solution (1.0 M solution in toluene) was added dropwise in an ice-bath. The solution became red brown. The solution was allowed to warm slowly to room temperature, and was stirred for 24 h. The solvent was evaporated under reduced pressure. The residue was then added to 10 mL hexane and stirred for 1 h, filtering and washing with hexane to obtain a white powder (0.20 g) in a yield of 49%. ^1^H NMR (400 MHz, C_6_D_6_, 25 °C): *δ* 8.40 (d, *J* = 8.0 Hz, 1H), 7.55 (d, *J* = 8.0 Hz, 1H), 7.50 (d, *J* = 8.0 Hz, 1H), 7.32 (t, *J* = 8.0 Hz, 1H), 7.22–7.19 (s, 1H), 7.02 (t, *J* = 8.0 Hz, 1H), 6.86–6.74 (m, 3H), 6.66 (d, *J* = 8.0 Hz, 1H), 6.46 (d, *J* = 8.0 Hz, 1H), 5.04 (s, 1H, NH), 3.20–3.14 (m, 1H), 2.55–2.50 (m, 2H), 1.59 (s, 3H), 1.40 (d, *J* = 8.0 Hz, 3H), 1.19 (t, *J* = 8.0 Hz, 3H), −0.08 (s, 3H), −0.66 (s, 3H). ^13^C NMR (100 MHz, C_6_D_6_, 25 °C): *δ* 145.9, 143.8, 141.7, 137.1, 134.4, 132.3, 130.1, 128.8, 127.6, 127.1, 126.5, 125.7, 125.6, 124.4, 123.5, 120.3, 79.7, 78.6, 50.9, 31.6, 24.3, 21.4, 13.6, −3.0, −8.2. Anal. calcd for C_54_H_60_Al_2_N_2_O_2_: C, 78.80; H, 7.35; N, 3.40. Found: C, 78.45; H, 7.31; N, 3.57.

#### Synthesis of Al2

In a manner similar to that described for Al1, using L2 instead of L1, Al2 was prepared as a white powder (0.63 g) in 35% yield. ^1^H NMR (400 MHz, C_6_D_6_, 25 °C): *δ* 8.41 (d, *J* = 8.0 Hz, 1H), 7.57 (d, *J* = 8.0 Hz, 1H), 7.51 (d, *J* = 8.0 Hz, 1H), 7.32 (t, *J* = 8.0 Hz, 1H), 7.21 (t, *J* = 8.0 Hz, 1H), 7.04 (d, *J* = 8.0 Hz, 1H), 6.79 (t, *J* = 8.0 Hz, 1H), 6.74 (s, 1H), 6.70 (s, 1H), 6.28 (s, 1H), 5.01 (s, 1H, NH), 3.20–3.14 (m, 1H), 2.56–2.50 (m, 2H), 1.97 (s, 3H), 1.59 (s, 3H), 1.43 (d, *J* = 8.0 Hz, 3H), 1.22 (t, *J* = 8.0 Hz, 3H), −0.07 (s, 3H), −0.62 (s, 3H). ^13^C NMR (100 MHz, C_6_D_6_, 25 °C): 145.6, 143.2, 138.0, 137.2, 136.5, 134.0, 131.9, 130.3, 128.7, 128.2, 127.9, 127.7, 127.3, 125.7, 124.3, 124.2, 123.9, 119.8, 79.9, 77.4, 50.5, 31.6, 24.3, 21.4, 14.0, −5.2, −9.9. Anal. calcd for C_56_H_64_Al_2_N_2_O_2_: C, 79.03; H, 7.58; N, 3.29. Found: C, 79.35; H, 7.41; N, 3.47.

#### Synthesis of Al3

In a manner similar to that described for Al1, using L3 instead of L1, Al3 was prepared as a white powder (0.14 g) in 32% yield. ^1^H NMR (400 MHz, C_6_D_6_, 25 °C): *δ* 8.34 (d, *J* = 8.0 Hz, 1H), 7.47 (d, *J* = 8.0 Hz, 2H), 7.29 (t, *J* = 8.0 Hz, 1H), 7.19 (d, *J* = 8.0 Hz, 1H), 7.03–6.88 (m, 3H), 6.79–6.74 (m, 2H), 6.50 (d, *J* = 8.0 Hz, 1H), 5.25 (s, 1H, NH), 3.13–3.03 (m, 1H), 1.69 (s, 3H), 1.66 (s, 3H), 1.32 (d, *J* = 8.0 Hz, 3H), 1.20 (d, *J* = 8.0 Hz, 3H), 0.66 (s, 3H), −0.13 (s, 3H), −0.67 (s, 3H). ^13^C NMR (100 MHz, C_6_D_6_, 25 °C): *δ* 144.7, 142.0, 141.8, 138.8, 137.9, 136.4, 134.9, 133.8, 129.3, 125.7, 124.9, 124.7, 123.9, 122.0, 121.6, 83.3, 51.1, 34.7, 30.0, 29.7, 29.2, 24.2, 22.7, 21.4, −4.1, −8.7. Anal. calcd for C_58_H_68_Al_2_N_2_O_2_: C, 79.24; H, 7.80; N, 3.19. Found: C, 79.45; H, 7.52; N, 3.24.

#### Synthesis of Al4

In a manner similar to that described for Al1, using L4 instead of L1, Al4 was prepared as a white powder (0.40 g) in 22% yield. ^1^H NMR (400 MHz, C_6_D_6_, 25 °C): *δ* 8.81 (d, *J* = 7.6 Hz, 1H), 7.45 (d, *J* = 7.6 Hz, 1H), 7.37 (d, *J* = 7.6 Hz, 1H), 7.29 (t, *J* = 7.6 Hz, 1H), 7.14–7.11 (m, 1H), 7.06–7.01 (m, 1H), 6.96 (t, *J* = 7.6 Hz, 1H), 6.81–6.77 (m, 2H), 6.68 (t, *J* = 7.6 Hz, 1H), 6.53 (d, *J* = 7.6 Hz, 1H), 6.36–6.34 (m, 1H), 5.14 (s, 1H, NH), 3.10–3.04 (m, 1H), 2.46–2.40 (m, 2H), 2.11–1.99 (m, 2H), 1.95–1.79 (m, 2H), 1.32 (d, *J* = 7.6 Hz, 3H), 1.12 (d, *J* = 7.6 Hz, 3H), 0.91–0.82 (m, 2H), 0.69 (t, *J* = 6.8 Hz, 3H), −0.08 (s, 3H), −0.60 (s, 3H). ^13^C NMR (100 MHz, C_6_D_6_, 25 °C): *δ* 142.0, 140.8, 136.4, 134.2, 132.4, 130.6, 129.3, 128.7, 128.6, 128.3, 127.5, 127.4, 125.7, 125.2, 123.5, 120.3, 79.4, 51.2, 41.3, 27.7, 24.4, 23.2, 23.1, 21.4, 14.0, 13.3, −1.95, −6.03. Anal. calcd for C_60_H_72_Al_2_N_2_O_2_: C, 79.44; H, 8.00; N, 3.09. Found: C, 79.56; H, 7.82; N, 3.18.

#### Synthesis of Al5

In a manner similar to that described for Al1, using L5 instead of L1, Al5 was prepared as a white powder (0.89 g) in 47% yield. ^1^H NMR (400 MHz, C_6_D_6_, 25 °C): *δ* 8.88 (d, *J* = 7.6 Hz, 1H), 7.49 (d, *J* = 7.6 Hz, 1H), 7.41 (d, *J* = 7.6 Hz, 1H), 7.33 (t, *J* = 7.2 Hz, 1H), 7.19 (d, *J* = 6.8 Hz, 1H), 7.00 (t, *J* = 7.6 Hz, 1H), 6.74 (t, *J* = 7.6 Hz, 1H), 6.70 (s, 1H), 6.61 (d, *J* = 7.6 Hz, 1H), 6.20 (s, 1H), 5.14 (s, 1H, NH), 3.12–3.07 (m, 1H), 2.50–2.44 (m, 2H), 1.97 (s, 3H), 1.38 (d, *J* = 7.6 Hz, 3H), 1.31–1.11 (m, 8H), 0.92 (t, *J* = 7.2 Hz, 3H), 0.72 (t, *J* = 6.8 Hz, 3H), −0.03 (s, 3H), −0.52 (s, 3H). ^13^C NMR (100 MHz, C_6_D_6_, 25 °C): *δ* 142.2, 139.1, 138.3, 137.1, 136.5, 134.2, 132.5, 130.3, 128.7, 128.3, 127.8, 127.6, 125.7, 125.2, 124.2, 120.4, 79.6, 51.2, 41.4, 32.0, 27.8, 24.4, 23.2, 23.1, 21.4, 14.4, 14.0, 13.5, −1.94, −5.82. Anal. calcd for C_62_H_76_Al_2_N_2_O_2_: C, 79.62; H, 8.19; N, 3.00. Found: C, 79.48; H, 8.22; N, 3.08.

#### Synthesis of Al6

In a manner similar to that described for Al1, using L6 instead of L1, Al6 was prepared as a white powder (0.22 g) in 11% yield. ^1^H NMR (400 MHz, C_6_D_6_, 25 °C): *δ* 8.84 (d, *J* = 7.6 Hz, 1H), 7.48 (d, *J* = 7.6 Hz, 1H), 7.36 (d, *J* = 7.6 Hz, 1H), 7.29 (t, *J* = 7.6 Hz, 1H), 7.13–7.11 (m, 1H), 7.06–7.00 (m, 1H), 6.98 (t, *J* = 7.6 Hz, 1H), 6.82–6.78 (m, 2H), 6.69 (t, *J* = 7.6 Hz, 1H), 6.54 (d, *J* = 7.6 Hz, 1H), 6.38–6.33 (m, 1H), 5.12 (s, 1H, NH), 3.15–3.08 (m, 1H), 1.67 (d, *J* = 7.6 Hz, 6H), 1.32 (d, *J* = 6.4 Hz, 3H), 1.20 (d, *J* = 6.8 Hz, 3H), 0.65 (s, 3H), −0.05 (s, 3H), −0.58 (s, 3H). The ^13^C NMR signals were not resolved due to the low signal to noise ratio. Anal. calcd for C_64_H_80_Al_2_N_2_O_2_: C, 79.80; H, 8.37; N, 2.91. Found: C, 79.64; H, 8.34; N, 3.12.

### General procedure for ε-caprolactone ring-opening polymerization

A typical ring-opening polymerization procedure of ε-CL in the presence of one equivalent of benzyl alcohol (Table 3, run 5) is presented as follows. The precatalyst Al1 (0.0082 g, 0.020 mmol) was dissolved in 2 mL of toluene in a Schlenk flask at room temperature. To this mixture, benzyl alcohol (0.020 mmol) in toluene solution was added and stirred at room temperature for 5 min. Then, the flask was placed in a temperature-controlled oil bath preheated at 100 °C for 5 min, and ε-CL (0.571 g, 5.0 mmol) was injected. The polymerization was terminated by the addition of glacial acetic acid (*ca.* 0.2 mL) into the reaction mixture until the solution was stirred for the described amount of time. The resulting viscous solution was diluted with dichloromethane, and then transferred into a beaker containing cold methanol (100 mL) with stirring. The resultant polymer was collected on filter paper, and dried under reduced pressure to give a white solid.

### X-ray crystallographic studies

Crystals of L2, L4, and L5 suitable for X-ray diffraction analyses were obtained from their heptane solution at room temperature overnight. Crystals of the complex Al1 suitable for the X-ray diffraction analyses were obtained from its toluene solution. X-ray studies were carried out on a Rigaku Saturn724 + CCD with graphite-monochromatic Mo Kα radiation (*λ* = 0.71073 Å) at 173 (2) K. Cell parameters were obtained by global refinement of the positions of all collected reflections. Intensities were corrected for Lorentz and polarization effects and empirical absorption. The structures were solved by direct methods and refined by full-matrix least squares on *F*^2^. All hydrogen atoms were placed in calculated positions. Structure solution and refinement were performed by using the SHELXL-2014 package.^21^ Within the structure refinement of complex Al1, there were free solvent molecules that had no influence on the geometry of the main compounds. Therefore, the SQUEEZE option of the crystallographic program *PLATON*^22^ was used to remove these free solvents from the structure. Details of the X-ray structure determinations and refinements are provided in Table S1.† Details of the hydrogen-bonding interactions of ligands (L2, L4 and L5) are listed in Table S2.†

## Conflicts of interest

There are no conflicts to declare.

## Supplementary Material

RA-011-D1RA01288F-s001

RA-011-D1RA01288F-s002
