# Supplementary material for: Bimetallic aluminum complexes bearing novel spiro-phenanthrene-monoketone/OH derivatives: synthesis, characterization and the ring-opening polymerization of ε-caprolactone
Source: RSC Adv. 2021 Apr 8;11(22):13274–81. doi: 10.1039/d1ra01288f (PMC8697580; doi:10.1039/d1ra01288f)
Supplement: RA-011-D1RA01288F-s001 [file RA-011-D1RA01288F-s001.pdf]

Electronic Supplementary Material (ESI) for RSC Advances.
This journal is © The Royal Society of Chemistry 2021

Bimetallic Aluminum complexes bearing the novel spiro-phenanthrene-monoketone/OH derivatives: synthesis, characterization and the ring-opening polymerization of *ε*-caprolactone

Erlin Yue, [a] Furong Cao, [b] Jun Zhang, [c] Wenjuan Zhang, * [b] Youshu Jiang, [c] Tongling Liang [c] and Wen-Hua Sun *[c]

[a] *Yan'an Key Laboratory of New Energy & New Functional Materials, Shaanxi Key Laboratory of Chemical Reaction Engineering, School of Chemistry and Chemical Engineering, Yan'an University, Yan'an 716000, China. E-mail: yueerlin@yau.edu.cn*

[b] *Beijing Key Laboratory of Clothing Materials R&D and Assessment, Beijing Engineering Research Center of Textile Nanofiber, School of Materials Science and Engineering, Beijing Institute of Fashion Technology, Beijing 100029, China. E-mail: zhangwj@bift.edu.cn*

[c] *Key Laboratory of Engineering Plastics and Beijing National Laboratory for Molecular Sciences, Institute of Chemistry, Chinese Academy of Sciences, Beijing 100190, China. E-mail: whsun@iccas.ac.cn*

Contents

FS1-FS10 ^{1}H and ^{13}C NMR spectra of complexes **Al1**−**Al5**

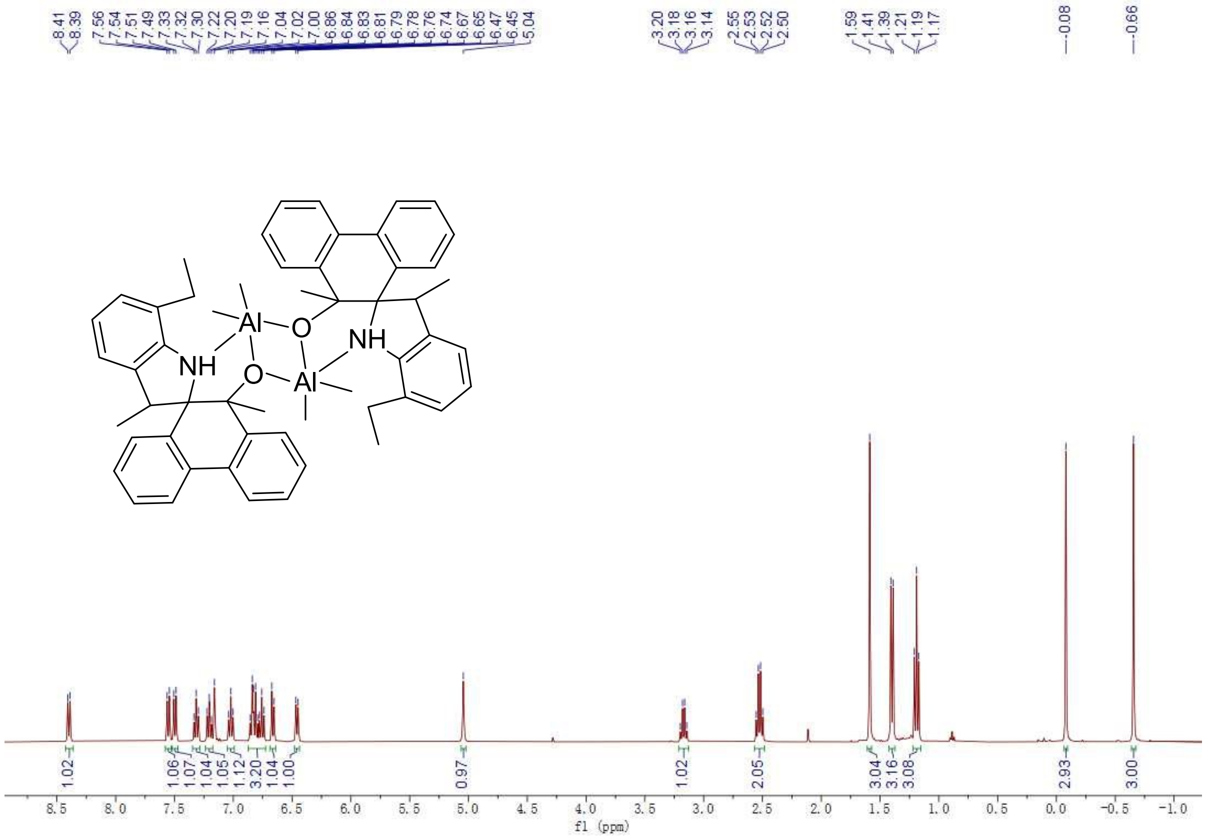

Figure S1 ^{1}H NMR spectrum of complex **Al1** (400 MHz, C_6D_6, 25°C)

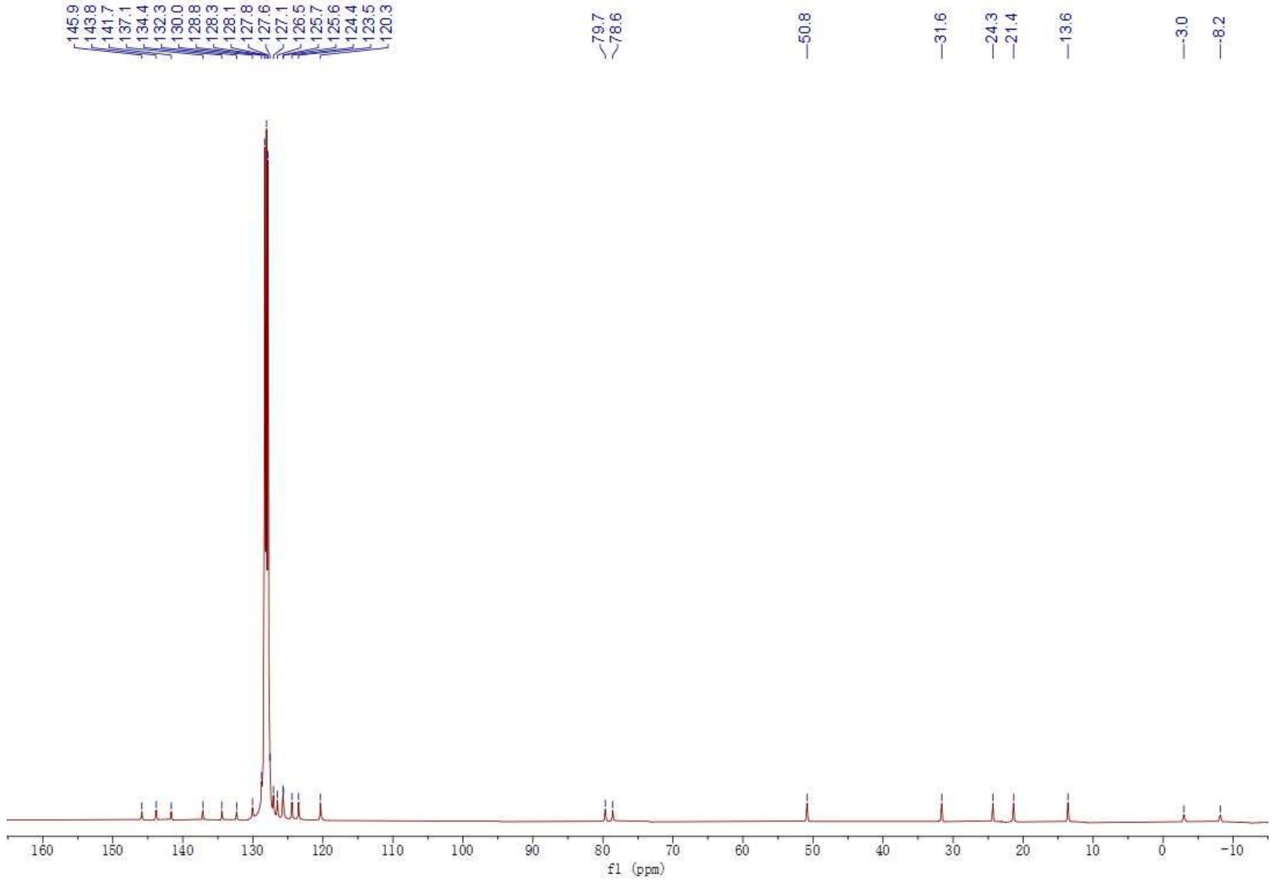

Figure S2 ^{13}C NMR spectrum of complex **Al1** (100 MHz, C_6D_6, 25°C)

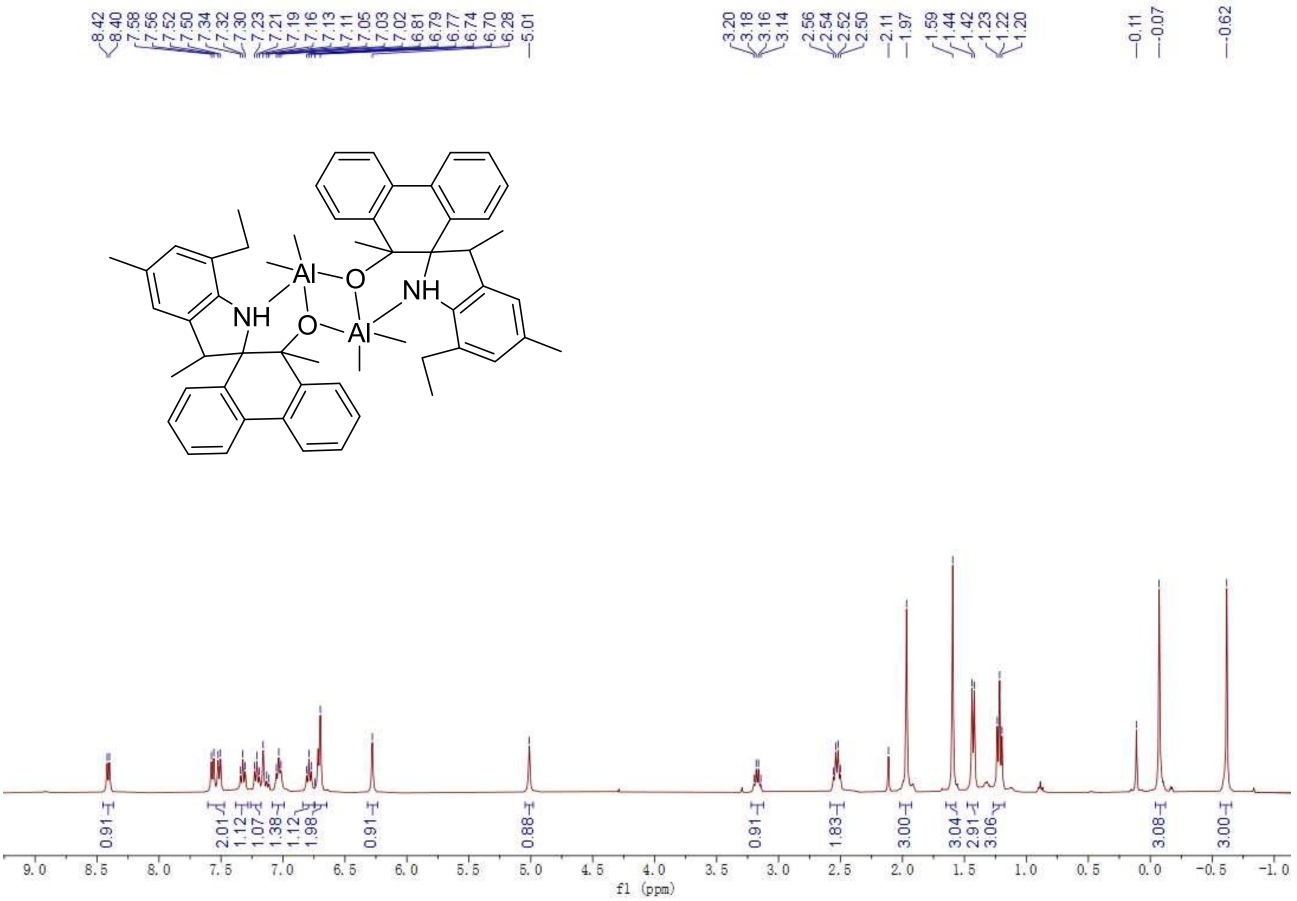

Figure S3 ^{1}H NMR spectrum of complex **Al2** (400 MHz, C_6D_6, 25°C)

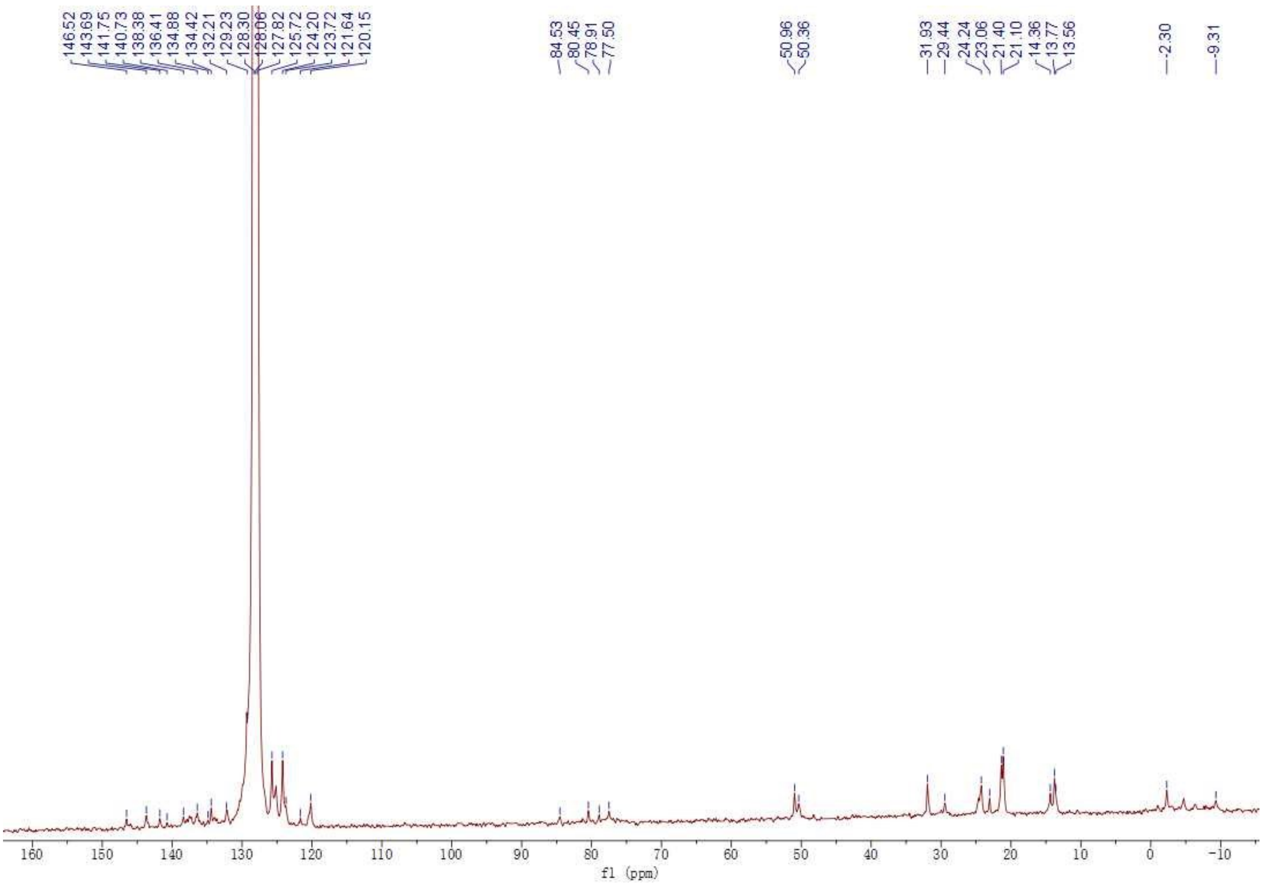

Figure S4 ^{13}C NMR spectrum of complex **Al2** (100 MHz, C_6D_6, 25°C)

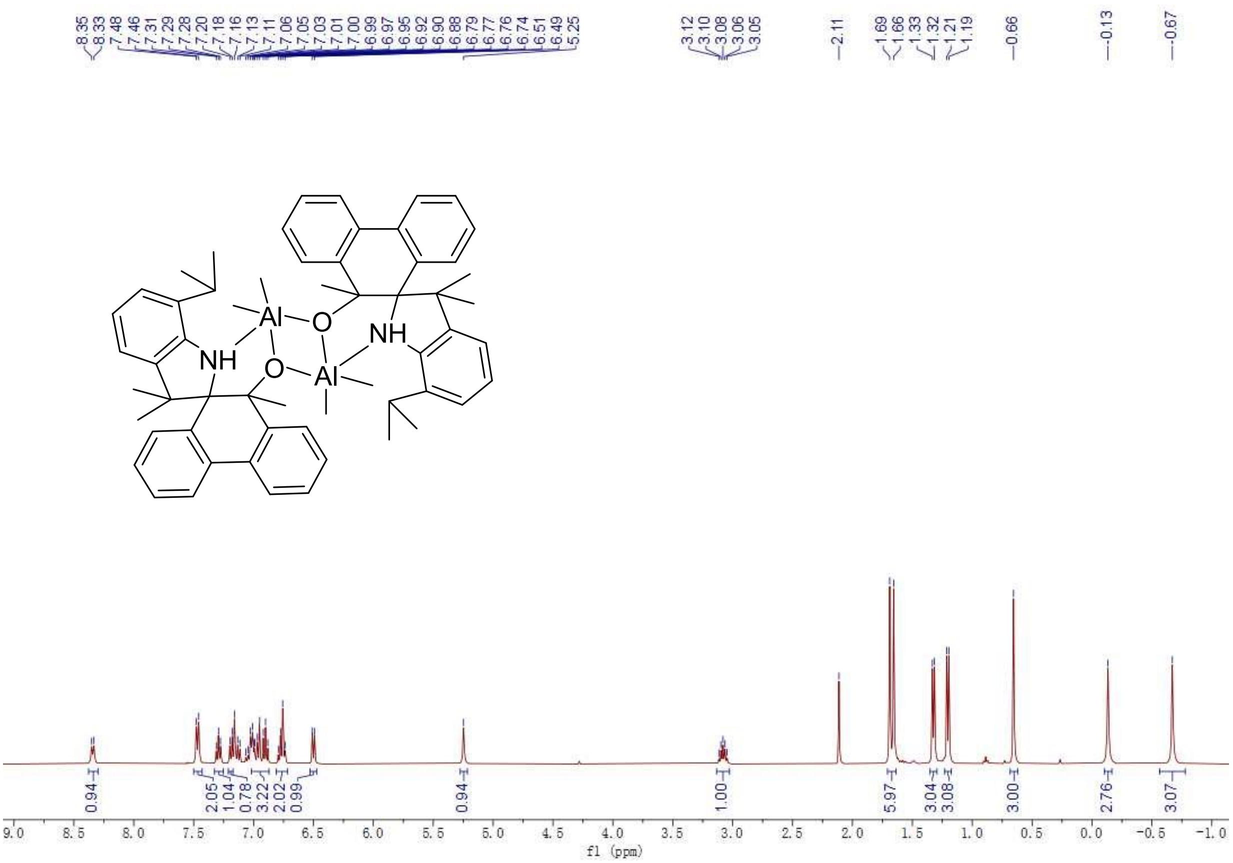

Figure S5 ^{1}H NMR spectrum of complex **Al3** (400 MHz, C_6D_6, 25°C)

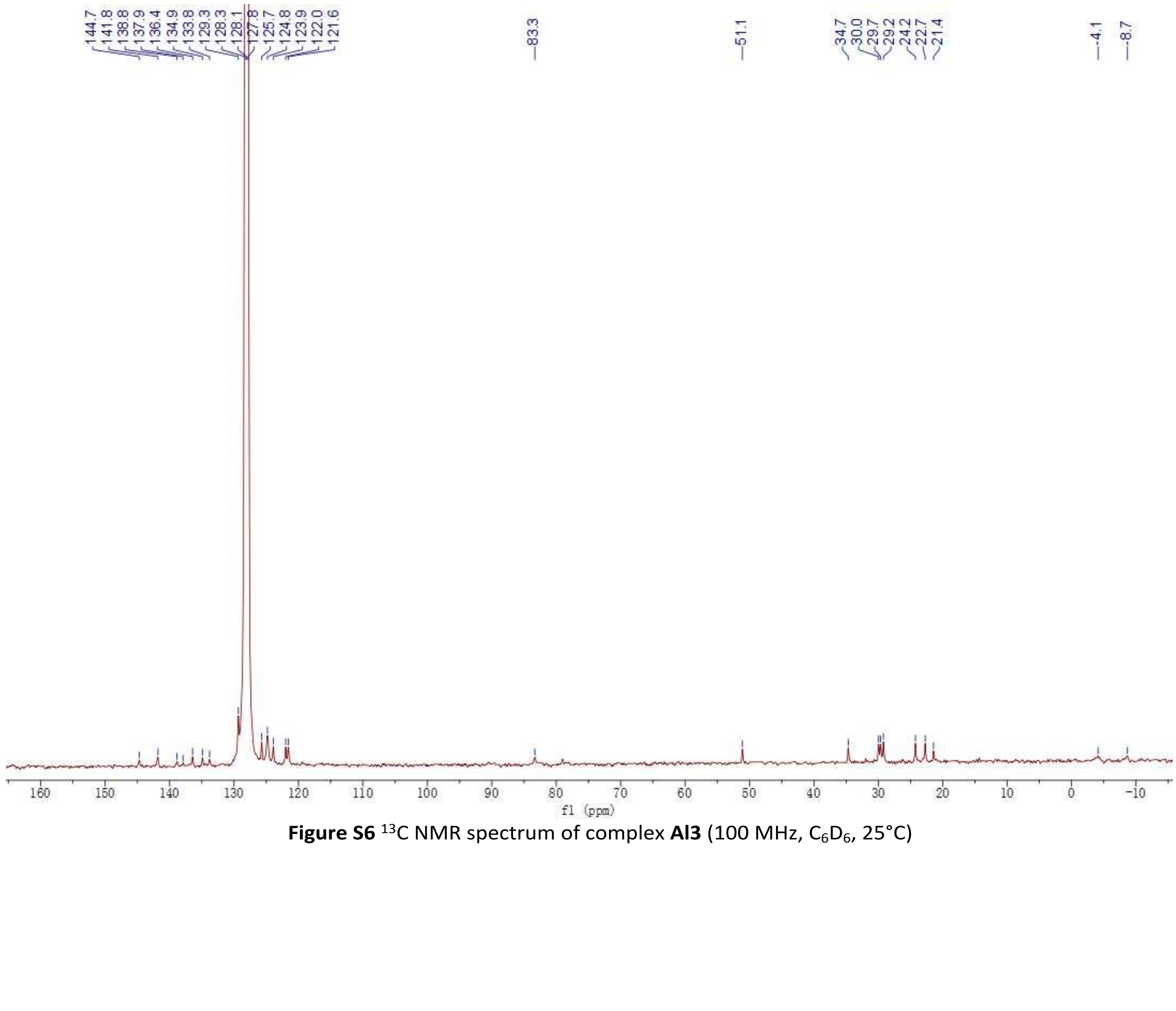

Figure S6 ^{13}C NMR spectrum of complex **Al3** (100 MHz, C_6D_6, 25°C)

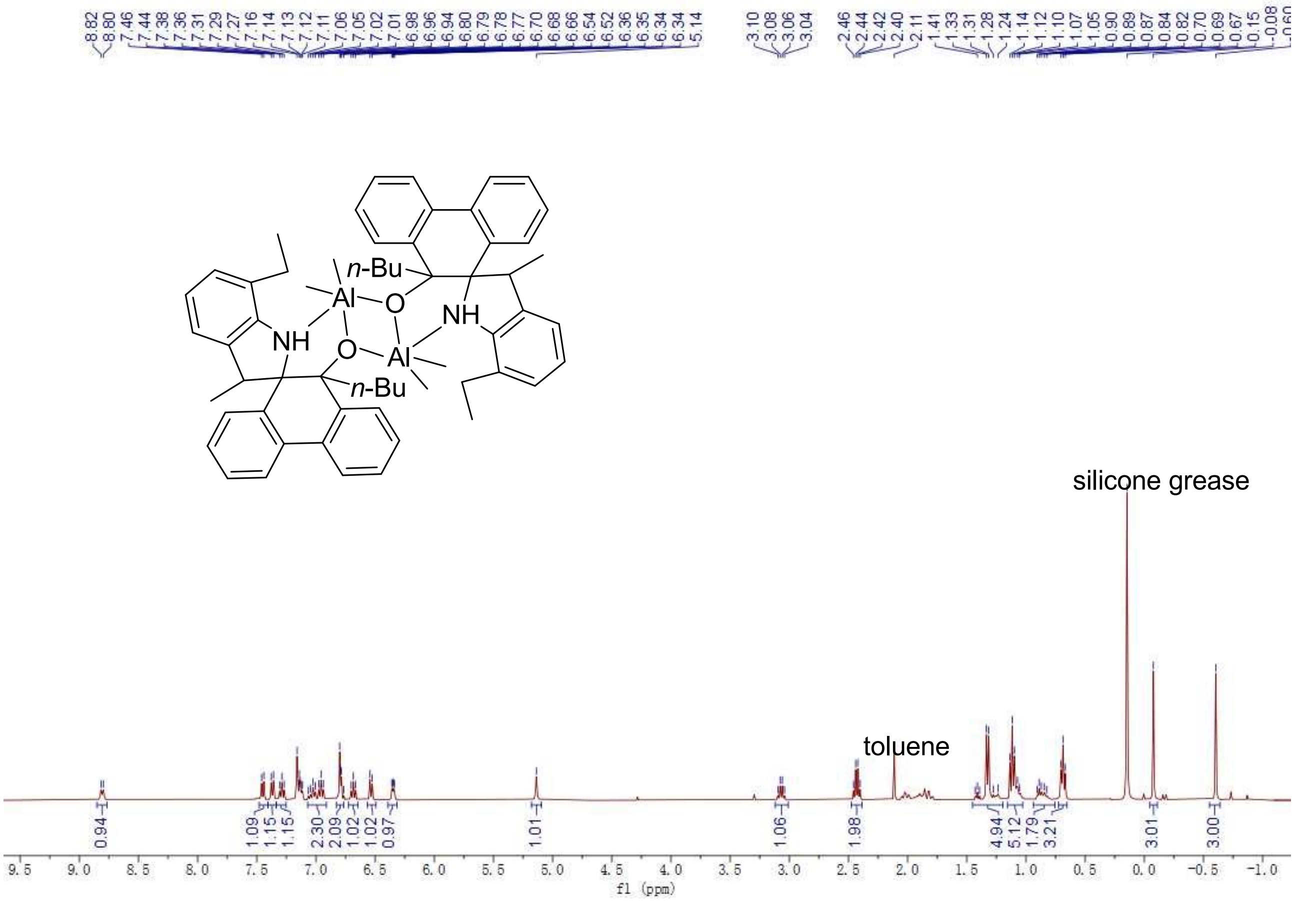

Figure S7 ^{1}H NMR spectrum of complex **Al4** (400 MHz, C_6D_6, 25°C)

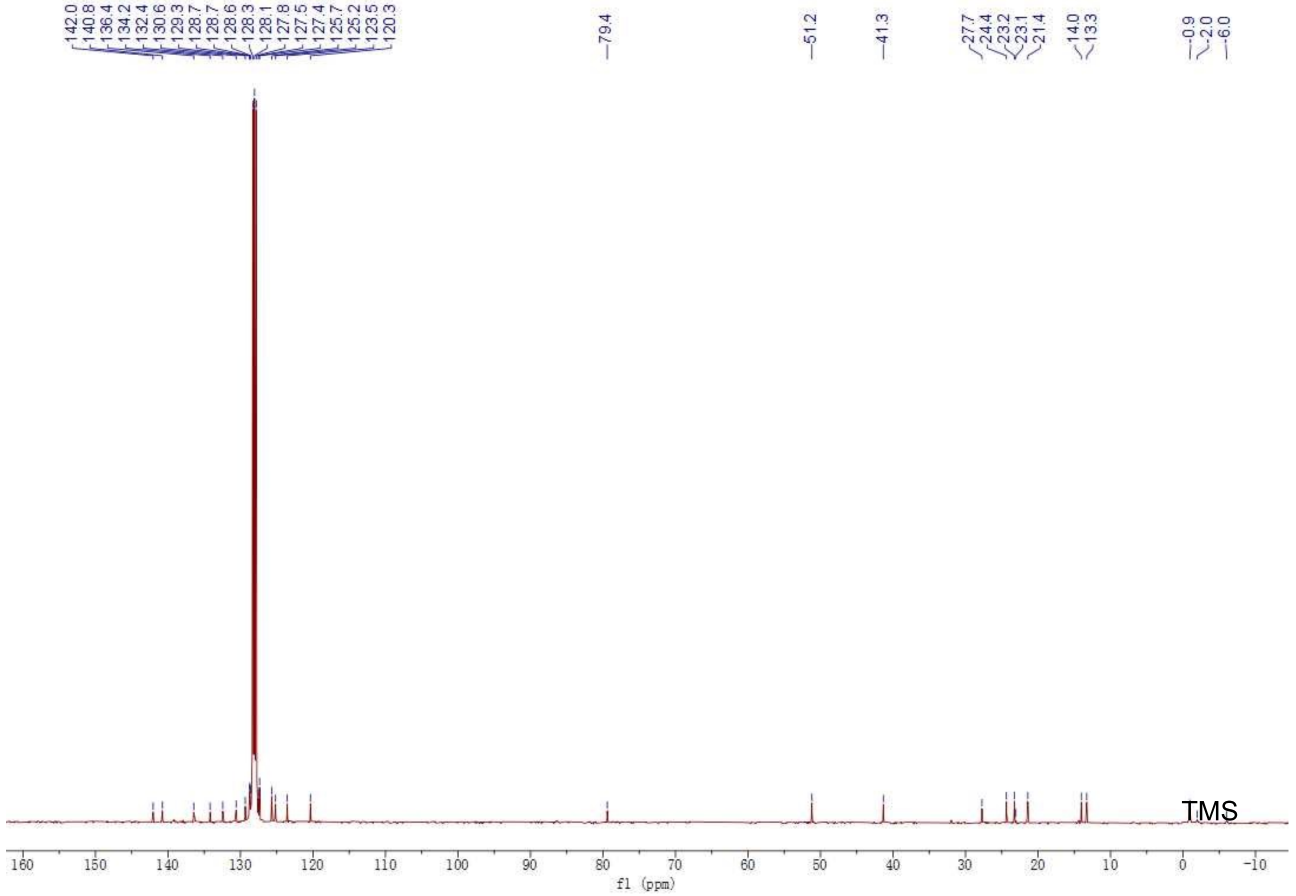

Figure S8 ^{13}C NMR spectrum of complex **Al4** (100 MHz, C_6D_6, 25°C)

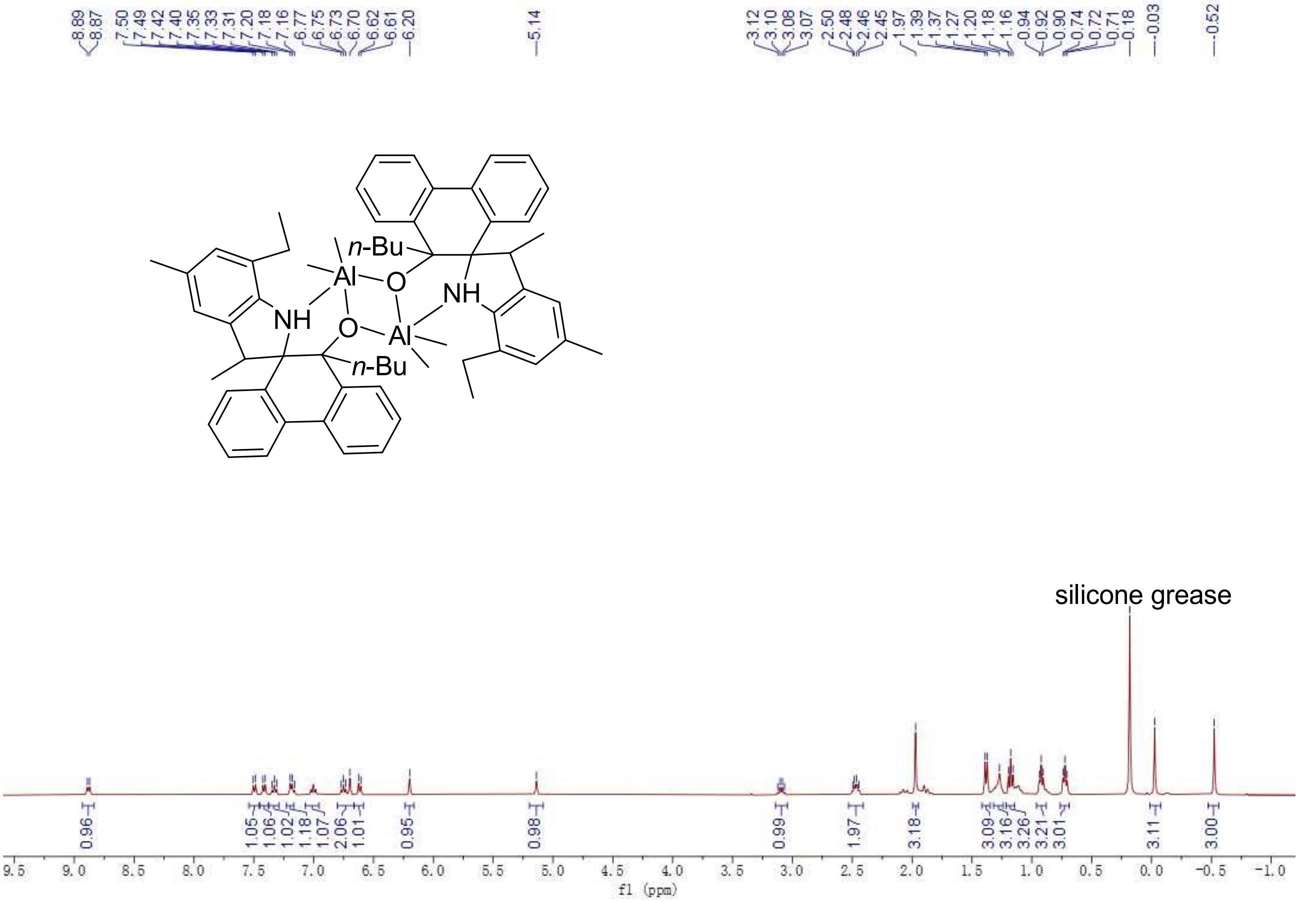

Figure S9 ^{1}H NMR spectrum of complex **Al5** (400 MHz, C_6D_6, 25°C)

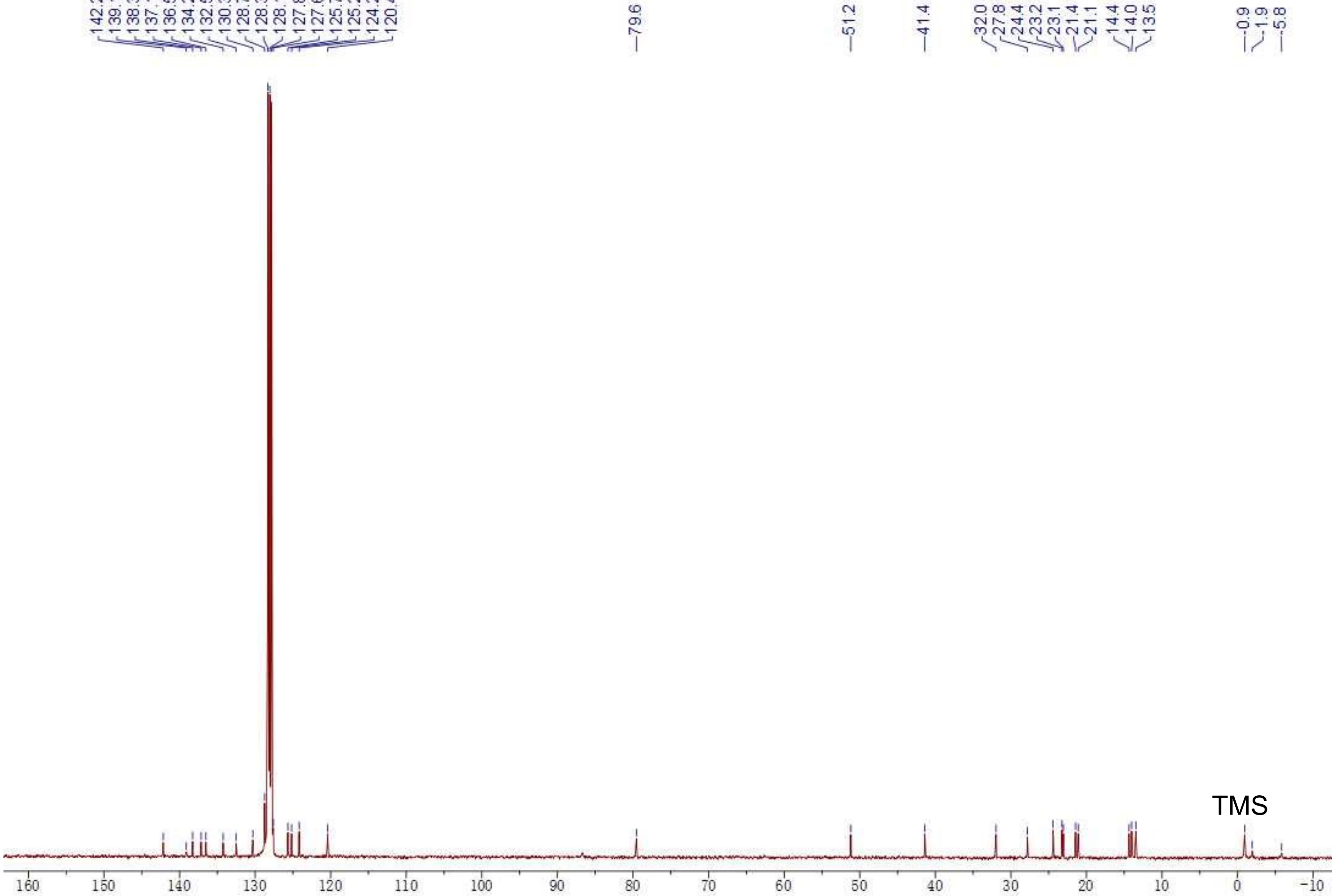

Figure S10 ^{13}C NMR spectrum of complex **Al5** (100 MHz, C_6D_6, 25°C)
